# Paradoxes in carcinogenesis: New opportunities for research directions

**DOI:** 10.1186/1471-2407-7-151

**Published:** 2007-08-06

**Authors:** Stuart G Baker, Barnett S Kramer

**Affiliations:** 1 Division of Cancer Prevention, National Cancer Institute, Bethesda, MD, USA; 2 Office of Disease Prevention, National Institutes of Health, Bethesda, MD, USA

## Abstract

**Background:**

The prevailing paradigm in cancer research is the somatic mutation theory that posits that cancer begins with a single mutation in a somatic cell followed by successive mutations. Much cancer research involves refining the somatic mutation theory with an ever increasing catalog of genetic changes. The problem is that such research may miss paradoxical aspects of carcinogenesis for which there is no likely explanation under the somatic mutation theory. These paradoxical aspects offer opportunities for new research directions that should not be ignored.

**Discussion:**

Various paradoxes related to the somatic mutation theory of carcinogenesis are discussed: (1) the presence of large numbers of spatially distinct precancerous lesions at the onset of promotion, (2) the large number of genetic instabilities found in hyperplastic polyps not considered cancer, (3) spontaneous regression, (4) higher incidence of cancer in patients with xeroderma pigmentosa but not in patients with other comparable defects in DNA repair, (5) lower incidence of many cancers except leukemia and testicular cancer in patients with Down's syndrome, (6) cancer developing after normal tissue is transplanted to other parts of the body or next to stroma previously exposed to carcinogens, (7) the lack of tumors when epithelial cells exposed to a carcinogen were transplanted next to normal stroma, (8) the development of cancers when Millipore filters of various pore sizes were was inserted under the skin of rats, but only if the holes were sufficiently small. For the latter paradox, a microarray experiment is proposed to try to better understand the phenomena.

**Summary:**

The famous physicist Niels Bohr said "How wonderful that we have met with a paradox. Now we have some hope of making progress." The same viewpoint should apply to cancer research. It is easy to ignore this piece of wisdom about the means to advance knowledge, but we do so at our peril.

## Background

Many scientists spend their careers performing studies designed to refine the knowledge base of existing paradigms of their science. Therefore much research is conservative in the sense that it tries to patch small holes in the existing paradigm, running the risk of ignoring gaping chasms. The prevailing paradigm in cancer research is the somatic mutation theory (SMT) that posits that cancer begins with a single mutation in a somatic cell followed by successive mutations [1]. Much current cancer research involves cataloging the ever increasing menagerie of genetic changes associated with cancer and trying to explain how they synergistically account for cancer phenotypes. This process of building ever more elaborate genetic models of carcinogenesis has been likened to adding epicycle models to the pre-Copernican Ptolemeic paradigm of planetary motion in order to explain discrepancies in astronomical data without postulating that the earth revolves around the sun [2]. The description of the motion of each newly discovered planetary body had to be retrofitted to Ptolemy's theory of "planetary perfection" [3]. While it is commendable to pursue a particular line of research to fill small cracks in theory, there is a problem when such a focus leads researchers to ignore the larger fissures that challenge the entire edifice. As we discuss, under SMT, there are paradoxical results involving initiation and promotion experiments, genetic instabilities, spontaneous regression, transplantation experiments, and foreign-body carcinogenesis. In our view ignoring these paradoxes misses a golden opportunity for new insights. We believe there should always be provision for "paradox-driven" research initiatives. In fact, the director of the U.S. National Institutes of Health, Dr. Elias Zerhouni, has recently initiated an innovation awards funding mechanism in this very spirit [4].

## Discussion

A result is paradoxical when the prevailing theory offers no likely explanation. When investigating reproducible, paradoxical results, it is helpful to consider alternative theories. One alternative to SMT is tissue organization field theory (TOFT) that posits that cancer arises from defects in cell communication often, but not necessarily, between the parenchyma, the functional tissue where most cancers arise, and the stroma, the surrounding connective tissue [2,5,6]. Results that are paradoxical under SMT may not be paradoxical under TOFT. Regardless of the validity of TOFT, the consideration of this alternate theory can be an important impetus for new research. To better illustrate how a paradox can lead to new study designs, we propose a microarray experiment to investigate the paradoxical results from experiments in plastic-film carcinogenesis.

### Paradoxes in initiation and promotion experiments

Initiation and promotion refer to observations about carcinogenesis in the following context: when investigators apply two particular treatments to the skin of rats at some time apart, the rats develop cancers at the location of the treatments; these localized cancers appear only after the application of the second treatment and do not develop when the order of treatments is reversed. The first treatment has been called the initiator and the second the promoter and the corresponding effects are called initiation and promotion. These experiments suggest that the initiator induces an irreversible persistent change [7]. Under the SMT the explanation is clear: the irreversible persistent change is a mutation of the genes in a skin cell. This explanation has become virtually axiomatic in the field of cancer research.

However some of these experiments reveal a paradoxical result under SMT, namely the presence of large numbers of spatially distinct precancerous lesions at the onset of promotion [8]. This phenomena is called field cancerization and its explanation is an important unanswered question under SMT [9]. One SMT explanation is that each precancerous lesion arises from a separate mutation; another SMT explanation is that a single genetic event occurs in a cell that clonally expands and migrates to other sites [9]. A TOFT explanation is that initiation creates an irreversible non-mutational change in the field of stromal tissue and promotion acts on the initiated stromal tissue to alter communication to many cells in the skin parenchyma. The current dominance of experiments on cell lines, tissue culture, and tumors locks the focus on the cancer cell, at the expense of surrounding stroma. Whatever the explanation, detailed study of these precancerous lesions would be an illuminating topic for additional research.

### Paradoxes in genetic instability in cancer and precancerous cells

It is well known that cancer cells exhibit numerous genomic alterations, which are sometimes called genetic instability. Under the SMT, the genetic mutations arise as part of a stochastic cascade of mutations on the way from an initial mutated cell to malignant cancer. The paradoxical results are the large number of genetic instabilities also found in benign neoplasms and hyperplastic polyps that are not considered cancers or even premalignant [10,11]. The most likely SMT explanation is multiple mutations, which is inconsistent with the generally accepted view that mutations are rare. A TOFT explanation is that the genetic instability of tumors is a consequence, not a cause, of cancer. One model consistent with TOFT postulates that the defect in cell communication directly alters gene expression leading to inactivated DNA segments that are not repaired under normal processes, yielding an accumulation of mutations [12]. This model makes biological sense because cells would not waste energy repairing inactivated DNA of little functional consequence [12]. Support for this model comes from experiments showing that repair of mutations caused by ultraviolet radiation was more efficient in expressed versus silent genes [12,13]. Support for the TOFT explanation also comes from experiments showing that initiation of cancer by an altered stromal environment resulted in genetic instabilities [14]. Trying to determine if genetic instabilities are a cause or an effect of cancer would make a fruitful research enterprise.

### Paradoxes in spontaneous regression

In rare cases, some tumors regress spontaneously. Under SMT, spontaneous regression might be explained by the extinction of cells carrying the mutation [15], but the details are not known. Under TOFT spontaneous regression can arise if the normal reciprocal intercellular signals are no longer disrupted. Because spontaneous regression is rare (frequent spontaneous differentiation of screening detected neuroblastoma of infancy is a notable exception), research on spontaneous regression in human cancer is difficult. However animal models and three dimensional cultures [16] could provide promising avenues for additional research along these lines. Also a related phenomenon, the replacement of the intraductal component of breast cancer by fibrous tissue, was observed in 21 of 311 patients undergoing breast conservation therapy [17]. A molecular study of these phenomena might yield promising leads.

### Paradoxes involving inherited mutations related to DNA repair

Fibroblasts from patients with xeroderma pigmentosa have a defect in repairing DNA damage that is either caused by some chemicals or ultraviolet light. If DNA damage were directly causing cancer, one would expect that exposure to ultraviolet light would increase the risk of skin cancer and exposure to chemicals in the environment would increase the risk of other cancers, so it is puzzling that patients with xeroderma pigmentosa have elevated rates of skin cancer but not elevated rates of other types of cancer [18]. Further investigation is also need to explain why patients with Cockayne syndrome and trichothiodystrophy have no increased rates of skin cancers although they have a comparable defect in DNA repair [19].

### Paradoxes involving Down's syndrome

Another paradoxical result is that persons with Down's syndrome are at much higher risk than the general population for leukemia and testicular cancer, but paradoxically at much lower risk for solid tumors, particularly breast cancer. When interpreting epidemiological studies that report incidence rates of many cancers, one must consider the possibility of finding an extreme cancer incidence rate by chance simply because incidence rates in many cancers were examined – the "multiple comparisons problem." Here the possibility of a chance result is diminished because the results are derived from three studies. The first study published in 2000 can be viewed as hypothesis generating; its main finding was that, among 2084 Danish persons with Down's syndrome, the incidence of leukemia was higher than the general population and the incidence of solid tumors (particularly breast cancer) was lower than in the general population [20]. A study published in 2002 involving 17897 Americans with Down's syndrome confirmed the higher incidence of leukemia and the lower incidence of solid tumors, particularly breast cancer; it also noted a higher incidence of testicular cancer (which had also been elevated in the previous study) [21]. A study published in 2006 involving 3581 persons in Finland with Down's syndrome also reported higher risk of leukemia and testicular cancer and lower risk of solid tumors than in the general population [22].

The striking aspect of the aforementioned results is that the solid   cancers, which have a lower incidence in people with Down's syndrome,   are surrounded by stromal cells while leukemia and testicular   cancers, which have a higher incidence in people with Down's   syndrome, are either devoid of stroma or have poorly developed stroma. These results, which have no obvious SMT explanation, point to a connection between the stroma and cancer incidence, which would be consistent with TOFT. Further experimentation, prompted by these results, has been proposed to study the extracellular components in patients with Down's syndrome in relationship to the formation of breast cancer [23].

### Paradoxes in transplantation carcinogenesis

Some transplantation experiments have involved normal cells transplanted to other parts of the body or next to stroma previously exposed to a carcinogen. When normal murine ovary tissue is transplanted to the spleen, many of the mice with transplanted tissues develop cancer [18]. When normal rat mammary epithelial cells were transplanted next to stroma exposed to a chemical carcinogen (after previously clearing out the local epithelial cells), cancer developed in the epithelial cells at a much higher rate than in controls [24]. Also, in vivo experiments have shown that when unirradiated murine epithelial cells were transplanted next to the irradiated stroma (after previously clearing out the epithelial cells), cancer developed in the unirradiated cells at a much higher rate than in controls [25]. Other transplantation experiments have involved cancer cells or cells exposed to carcinogen transplanted to other parts of the body. When mouse teratocarcinoma cells were transplanted to normal mouse embryo, they had stable differentiation and were incorporated into the tissue [26]. When murine skin epithelial cells exposed to a carcinogen that would normally cause tumors in a few weeks were transplanted to an untreated site, no tumors developed [27]. SMT does not offer a likely explanation but a TOFT explanation of altered communication from the stroma to the parenchyma is clear. This  active area of research should be pursued. Microarray studies (as discussed in the next section) could be useful tools to try to understand the mechanism underlying these results.

### Paradoxes in foreign-body carcinogenesis

The discovery that materials inserted subcutaneously in animals can induce cancer was found serendipitously in the 1940's and was confirmed in experiments in the 1950's and 1960's. A primary finding was a similar carcinogenic response for various substances (including highly unreactive materials such as gold, platinum, and polyethylene) inserted subcutaneously, but only when they were implanted intact and not in a powdered form [28].

Lest one think that these experiments are artificial, there is speculation that the same mechanism could be responsible for the development of gallbladder cancers, which are strongly associated with the presence of gallstones, lung cancer arising near scar tissue ("scar cancers"), and lung cancer in smokers. Regarding the latter, in 1962, Passey wrote "my heresy is to believe that the excess of mucoid secretion, so often found in respiratory disease, is the condition responsible for many lung cancers. Mucus is sticky and will cling to a patch of respiratory epithelium for longer periods: by its blanketing action – occlusion – it will interfere with the normal exchange of gases and cellular fluids in the underlying cells" [29].

Our primary focus is on a 1973 study of morphological changes associated with the insertion of a subcutaneous Millipore filter in mice [30]. The salient aspects of the experiment are as follows. The investigators inserted into each mouse a Millipore filter 2 cm in diameter with a given pore size. The results clustered into two distinct groups. In what we call the small-pore group, the fraction of mice with the sarcomas was 11/11, 6/10, and 8/10 for implant pore sizes of 0.025, 0.05, and 0.10 μm respectively. In the large-pore group no sarcomas developed in groups of 9 mice with implant pore sizes of 0.45, 0.80, 3.00 and 8.00 μm, and 1 sarcoma arose in a group of 8 mice with pore size 3 μm and different (nylon reinforced) type of implant. To investigate morphology, the investigators killed additional mice at either 1, 3, 5, and 10 months after implantation and made the following observations. In the large-pore group, the filter was invaded by inflammatory macrophages or their cytoplasmic processes, and there was extensive intercellular contact throughout the filter by cytoplasmic processes. In the small-pore group, there was no invasion of the filter by cytoplasmic processes and thick fibrous capsules developed around implants creating a sharp demarcation between normal cells and the Millipore filter. Also in the small-pore group, an early anaplastic sarcoma was detected at 10 months and tumors developed by 22 months.

The main paradoxical result is the high incidence of sarcomas in the small-pore group and the negligible incidence of sarcomas in the large-pore group. That these two groups are distinct is confirmed by the presence or absence of invasion by cytoplasmic processes. These results are paradoxical under SMT because there is no obvious mechanism for an association between pore size and a genetic alteration.

There are two types of questions posed by these results (summarized in Table 1). The first question is what aspect of the difference in pore size is responsible for the differences in the incidence of sarcomas? The second is what is the cellular mechanism underlying the induction of sarcomas by Millipore filter? We discuss a possible experiment to address each question. The proposed experiments cannot definitively determine the validity of any particular theory. However it is hoped that it will provide new leads for future investigation and spur bench scientists to consider more studies outside the "box" of SMT.

**Table 1:** Possible experiments to investigate paradox associated with sarcomas and Millipore filters

Question	Proposed experiment	Rationale
What aspect of the implant is responsible for sarcomas?	2 × 2 × 2 × 2 factorial design involving surface area, roughness, electrostatic charge, pore size	A factorial design allows investigation of many factors at once.
How does the implant affect cellular changes?	Primary study: no implant versus small pore implant that induces sarcoma	Looking for salient differences in gene expression using multiple random validation and signatures with few genes
	Secondary study: no implant versus large pore implant that does not induce sarcoma	Control study to determine if differences in gene expression could reliably be associated with histological changes

#### What aspect of pore size is responsible for changes in incidence of sarcomas?

Before discussing an experiment, we discuss four possible explanations that have been offered. One possibility is that the incidence of sarcomas is related to the roughness of the material, which increases with pore size. However, despite noting that other experiments demonstrated an association between roughness and sarcoma incidence, the investigators concluded that for their experiment "this increase [in roughness] is gradual with only a slight difference in roughness between 0.22 and 0.1 μm ...This hardly explains the complete transition from negative to positive tumorigenicity in that range." A second possibility is that the incidence of sarcomas is related to surface area of the filter, which is the area of the filter inside the pores and hence approximately proportional to pore size. Again this explanation does not explain the sudden change from no incidence of tumors to high tumor incidence between filters with pore sizes of 0.22 and 0.1 μm; also it does not explain other experiments that found no sarcomas when inserted material was a powder. A third possibility is that increased incidence in the incidence of sarcomas could be related to changes in electric surface charges with to the pore size. However the investigators noted that "since ...the tumorigenic and nontumorigenic filters were hydrophilic and electropositive, the electric charge cannot generally be considered the major determining factor in foreign body carcinogenesis." A fourth possibility is that the Millipore filters disrupted cell communication when the holes were sufficiently small (a maximum of 0.10 μm) to impede the transfer of critical molecules between cells. Because sarcomas are cancers of the stroma, this explanation does not fit the theory of stromal cells affecting epithelial cells. However it is possible that the small pore size filters could have blocked vital communication from one part of the stroma to other.

Although the aforementioned evidence points to pore size alone, without its impact on charge, surface area, or roughness, as the most likely explanation for differences in incidences of sarcomas, a more definitive study could be conducted. A potential study design is a 2 × 2 × 2 × 2 factorial experiment involving 128 mice with the following four factors: pore size (0.025 μm versus 0.45 μm), roughness (two levels, perhaps either roughening or no roughing of the filter surface or a smooth or rough plastic lattice overlay related to [31]), surface area (either a single filter or a stacked pair of filters as in [32]), and electrostatic charge (two levels, perhaps hydrophobic versus normal filters as in [33]), The sample size of 8 per each of 16 groups is designed to an detect extreme interactions, namely a comparison between 9/10 in one group versus 1/10 in another group with power of .80 and Bonferroni type I error of .05/15, for 15 comparisons versus a reference group. However we recommend analysis via logistic regression to more systematically evaluate the factors.

#### What is the cellular mechanism underlying sarcomas induced by Millipore filters?

If researchers could understand the cellular mechanism by which subcutaneous implants induce sarcomas without any clear indication of a genetic change it would likely greatly help in making progress toward understanding carcinogenesis. Most of these experiments were conducted decades ago. Since then biotechnology has improved immensely.

We propose applying modern microarray technology to study the changes associated with carcinogenesis in the Millipore experiments involving different pore sizes. A possible experiment is the following. Three hundred mice would be randomized to three groups of 100 each, either controls with no implants, implants of Milllipore filters with small pores that yield high rates of sarcoma, and implants of Millipore filters with large pores that yield virtually no sarcomas. This sample size is larger than the size of other microarray studies (per group) that have identified genes that contribute strongly to good performance. From each mouse, investigators would take a sample of subcutaneous tissue from the site of the implant (or corresponding site in control) at 5 weeks.

The primary analysis would involve comparing the gene expression between controls and mice with filter implants having small pores. The goal is to find the major changes associated with the incidence of sarcoma. To this end, a conservative analysis using multiple random validation [34] is proposed. This method involves multiple random splits of the training and test samples. On each random split, a gene signature (classification rule) is selected in the training sample and its classification performance is evaluated in the test sample. There are mathematical reasons why most performance gain comes with the first few genes included in the signature [35]. Therefore it is recommended that performance be compared for signatures involving 1, 2,3, 5 and 10 genes to determine the signature length such that good classification performance is obtained and further gains in classification performance with longer signatures are minor [33]. For the selected gene signature, the histogram of genes selected on different random training sets would be plotted to determine if any genes are highly reproducible. The finding of highly reproducible genes could provide an important lead to the cellular mechanism underlying filter induced carcinogenesis [36].

A secondary analysis would be performed comparing the no implant group with a group receiving implants with large pores. This analysis serves as a control to determine if changes in gene expression could reliably be associated with histological changes and to determine if there were common cellular changes regardless of pore size.

## Summary

Paradoxical results are not uncommon in studies of carcinogenesis. Ignoring these paradoxes is tantamount to saying the prevailing theory holds in all instances except the paradoxical cases. However ignoring "outliers" in data analysis is not satisfying; it should be the last refuge when all else fails. But more importantly, ignoring paradoxical results means missing potentially exciting new avenues for research. Rather than relegate the paradoxical results to the periphery of investigations, they should be the centerpiece of a paradox-driven research portfolio.

## Competing interests

The author(s) declare that they have no competing interests.

## Authors' contributions

SGB wrote the initial draft and BSK provided substantive comments and substantial editing.

## Pre-publication history

The pre-publication history for this paper can be accessed here:


